# Roles of *NlAKTIP* in the Growth and Eclosion of the Rice Brown Planthopper, *Nilaparvata lugens* Stål, as Revealed by RNA Interference

**DOI:** 10.3390/ijms160922888

**Published:** 2015-09-22

**Authors:** Peiying Hao, Chaofeng Lu, Yan Ma, Lingbo Xu, Jiajun Zhu, Xiaoping Yu

**Affiliations:** Zhejiang Provincial Key Laboratory of Biometrology and Inspection & Quarantine, College of Life Sciences, China Jiliang University, Hangzhou 310018, China; E-Mails: lcf19890107@163.com (C.L.); ma_yan1989@126.com (Y.M.); 06c2800014@cjlu.edu.cn (L.X.); chuh2010@163.com (J.Z.)

**Keywords:** brown planthopper, AKT interacting protein, RNA interference, growth, eclosion, ovary development

## Abstract

AKT-interacting protein (AKTIP) interacts with serine/threonine protein kinase B (PKB)/AKT. AKTIP modulates AKT’s activity by enhancing the phosphorylation of the regulatory site and plays a crucial role in multiple biological processes. In this study, the full length cDNA of *NlAKTIP*, a novel AKTIP gene in the brown planthopper (BPH) *Nilaparvata lugens*, was cloned. The reverse transcription quantitive PCR (RT-qPCR) results showed that the *NlAKTIP* gene was strongly expressed in gravid female adults, but was relatively weakly expressed in nymphs and male adult BPH. In female BPH, treatment with dsAKTIP resulted in the efficient silencing of *NlAKTIP*, leading to a significant reduction of mRNA levels, about 50% of those of the untreated control group at day 7 of the study. BPH fed with dsAKTIP had reduced growth with lower body weights and smaller sizes, and the body weight of BPH treated with dsAKTIP at day 7 decreased to about 30% of that of the untreated control. Treatment of dsAKTIP significantly delayed the eclosion for over 7 days relative to the control group and restricted ovarian development to Grade I (transparent stage), whereas the controls developed to Grade IV (matured stage). These results indicated that *NlAKTIP* is crucial to the growth and development of female BPH. This study provided a valuable clue of a potential target *NlAKTIP* for inhibiting the BPH, and also provided a new point of view on the interaction between BPH and resistant rice.

## 1. Introduction

The brown planthopper (BPH) *Nilaparvata lugens* is a major insect pest for rice crops. In recent years, this pest has caused heavy losses in the rice yields in Asian countries [1,2,3]. The two main methods of controlling this pest are spraying chemical pesticides and using rice varieties that are BPH-resistant [4,5]. However, with the overuse of pesticides and the large cultivation areas of resistant rice varieties, some BPH colonies have developed strong adaptability to pesticides and/or resistant rice varieties [6,7]. Therefore, sustainable control of BPH is a serious problem, and to solve it requires exploring the interaction between BPH and resistant rice varieties, which includes revealing the molecular mechanism of BPH growth and development.

The AKT gene originates from the cellular homolog of the v-akt oncogene transduced by AKT8, a retrovirus isolated from a spontaneous thymoma cell line derived from AKR mice [8]. AKT is a serine/threonine protein kinase (also known as protein kinase B, PKB), and it is a critical cell signaling node that regulates a range of physiological processes [9]. The AKT signaling pathway plays a role in the regulation of cell growth, protein synthesis, antiapoptotic and survival signals [10,11,12]. In *Drosophila*, AKT regulates growth by promoting the insertion of glucose transporters into the cell membrane, inhibiting Forkhead box, Class O (FOXO), and inhibiting the Tuberous Sclerosis dimer TSC1/TSC2 [13,14,15]. In *Anopheles stephensi*, AKT signaling increase resulted in reduced mosquito lifespan [16]. Furthermore, activation of an AKT signaling cascade leads to the long-winged morph of BPH [17].

AKTIP (AKT interacting protein) is a membrane protein that can regulate the activity of AKT by enhancing the phosphorylation of the AKT protein, thereby acting as a regulator of the AKT signal pathway [18]. AKTIP is most commonly known as Ft1, and was initially identified in a mouse mutation called Fused toes (Ft), which involves defects in limb development [19]. Ft1 is conserved among mammals and a homologous gene is present in humans [20]. Depletion of the Ft gene in human embryonic kidney 293T cells affects the trafficking of the epidermal growth factor in early-to-late endosome/lysosomes [21]. Moreover, Ft1 has a strong effect on the apoptosis susceptibility of T lymphocytes treated with glucocorticoids [22]. Although AKTIP as well as Ft has been well studied in mammals, they are poorly understood in insects, and the exact function remains unknown in BPH.

Previously, we analyzed BPH gene expression profile [23] and found that a gene encoding AKT interacting protein (*NlAKTIP*) in our Rh colony [reared on resistant Rathu Heenati (RHT, with *Bph 3*) rice stain] was downregulated 5-fold compared with that in our Tn colony (reared on susceptible Taichung Native 1 (TN1, with no resistance gene) rice stain). At the same time, the mRNA expression level of most related genes in the AKT signaling pathway changed accordingly, suggesting that *NlAKTIP* might be an important responsive gene in BPH that feed on resistant rice. We therefore selected *NlAKTIP* for further analysis since it can affect the activity of AKT, a focal point of the AKT signaling pathway. We intended to explore the function of *NlAKTIP* in BPH and expected our study to provide new clues for screening important genes as targets for pest control.

## 2. Results

### 2.1. Cloning of NlAKTIP, Sequence Comparison and Phylogenetic Analysis

Full-length *NlAKTIP* cDNA (GenBank Acc no: KP027545, not released) was cloned using the rapid amplification of cDNA ends (RACE) method according to our previous transcriptome sequence information. The cloned full-length *NlAKTIP* cDNA was 964 bp, with an open reading frame (ORF) of 588 bp (Figure S1). This ORF encodes a 195-amino acid polypeptide with a calculated molecular mass of 22.19 kDa and a theoretical isoelectric point of 7.69. This polypeptide is similar to the ubiquitin ligase domain of other ubiquitin-conjugating enzymes but lacks the conserved cysteine residue, which is a characteristic of AKTIP. Therefore, this polypeptide may not be able to conjugate ubiquitin to the target protein, but it should conjugate with the AKT protein kinase and modulate its activity by enhancing phosphorylation [22].

**Figure 1:**
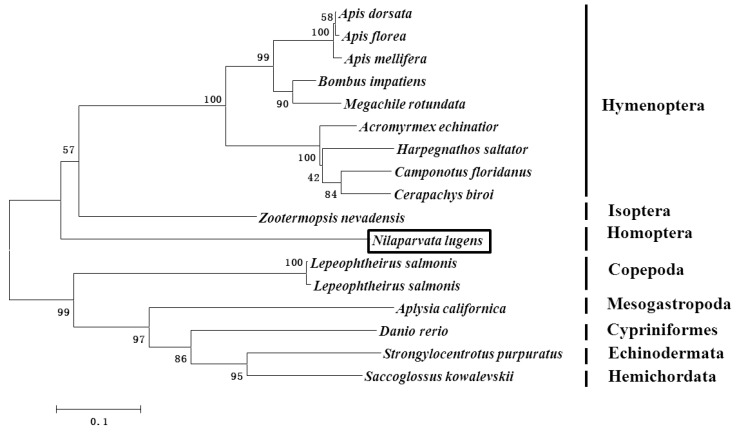
Phylogenetic analysis of AKT-interacting protein (AKTIP) in insects and other organisms. The phylogenetic analysis was constructed using the neighbor-joining method. Branch lengths are proportional to the sequence divergence. The bar represents 0.1 substitutions per site. The bootstrap values are shown in the nodes. The boxed *Nilaparvata lugens* was the insect of brown planthopper used in this study.

The phylogenetic analysis showed that *NlAKTIP* belongs to a single branch and has a large genetic distance from other organisms used in the phylogenetic analysis. In the phylogenetic analysis, the most closely related amino acid sequence to AKTIP (54% identity) was from *Zootermopsis nevadensis* (isoptera) (Figure 1).

### 2.2. The mRNA Expression of NlAKTIP in Different Colonies and Different Developmental Stages

**Figure 2:**
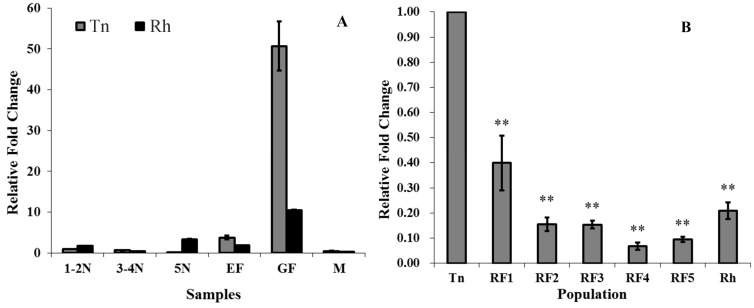
*NlAKTIP* mRNA expression in BPH. (**A**) Expression patterns of *NlAKTIP* mRNA at different developmental stages of the Tn and Rh colonies. The mRNA level of *NlAKTIP* was detected using RT-qPCR. The mRNA level was normalized relative to the *β-actin* transcription levels and the reference was the mRNA level of 1st–2nd Tn instar nymph. The results are expressed as the means ± S.E from three replicates. 1–2N: 1st–2nd instar nymph; 3–5N: 3rd–4th instar nymph; 5N: 5th instar nymph; EF: newly eclosed female; GF: gravid female, M: male; (**B**) *NlAKTIP* expression in the gravid adult female. The mRNA level of *NlAKTIP* was detected using RT-qPCR. The mRNA level was normalized relative to the β-actin transcription levels and the reference was the mRNA level of the Tn gravid adult female. Asterisks represent significant differences between reference Tn and other subjects (Student’s *t*-test, ** *p* < 0.01). The results are expressed as the means ± SE from three replicates.

The expression of *NlAKTIP* mRNA was investigated in the Tn colony (reared on susceptible TN1 rice) and Rh colony (reared on resistant RHT rice) by using reverse transcription quantitive PCR (RT-qPCR) at different developmental stages. The results showed that the *NlAKTIP* transcription level was relatively low in all nymph stages and in the adult male stage. The transcription level in the gravid female was the highest in both the Tn colony and the Rh colony (Figure 2A).

After the BPH was transferred from susceptible TN1 to resistant RHT, *NlAKTIP* mRNA expression levels in the gravid female tended to decrease compared with the Tn colony. As for RF strains (the Tn colony was exposed to resistant RHT for 1, 2, 3, 4, and 5 generations, respectively), the mRNA expression level of RF1 was 53.42% of that in the Tn clone, and showed a degree of difference from Tn (*p* = 0.013). In the RF2 and Rh colonies, the mRNA level of *NlAKTIP* significantly decreased to 4.46% and 27.88% (Figure 2B).

### 2.3. Effects of NlAKTIP Knockdown on mRNA and Protein Expression

**Figure 3:**
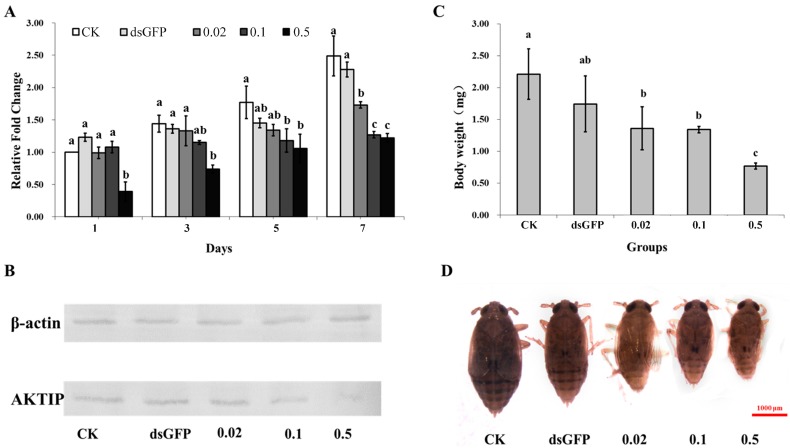
Effects of *NlAKTIP* RNAi. CK: female fed on D-97 (untreated diet); dsGFP: female fed on 0.5 μg/μL dsGFP control; 0.02: female fed on 0.02 μg/μL dsAKTIP; 0.1: females fed on 0.1 μg/μL dsAKTIP; 0.5: female fed on 0.5 μg/μL dsAKTIP. (**A**) *NlAKTIP* mRNA levels after feeding D-97, dsGFP or dsAKTIP. The results are expressed as the means ± SD from 20 individuals with three replications. The relative gene expression level was compared by Tukey’s test at each sampling time, and different letters of a, b, and c on the bars indicated significant differences among treatments (*p* < 0.05); (**B**) Western blot analysis of AKTIP from the different treatments; (**C**) Average body weights of different groups at day 7. The results are expressed as the means ± SD from at least 7 individuals. Symbols of a, b, and c indicate significant differences between each two groups (Student’s *t*-test, *p* < 0.05); (**D**) The effect of *NlAKTIP* RNAi on the adult female body size.

After RNAi was initiated by feeding the insects an artificial diet containing dsRNA, and samples were taken every two days when the BPH of the untreated control group began to eclose. The RT-qPCR results showed that the mRNA abundance of *NlAKTIP* increased from day 1 to day 7 in all groups (Figure 3A). However, dsAKTIP RNAi inhibited the expression of *NlAKTIP*, resulting in lower AKTIP mRNA abundance in dsAKTIP treated groups. RNAi with 0.5 μg/μL dsAKTIP resulted in the efficient silencing of *NlAKTIP*, leading to a significant reduction of mRNA levels to about 50% of that of the untreated control group at day 7. The knockdown effect was significantly correlated with the concentration of dsAKTIP. For example, at day 7, the expression levels of the target gene in the groups treated with medium (0.1 μg/μL) and high (0.5 μg/μL) concentrations of dsAKTIP were lower than that of the group treated with low (0.02 μg/μL) concentrations of dsAKTIP, as well as that of the untreated and GFP control groups. The expression level of the target gene in the low concentration group was moderate, but still significantly different from the untreated and the GFP groups. There were no significant differences between the untreated control group and GFP control group.

The effect of *NlAKTIP* knockdown was further demonstrated at protein level. Western blot analysis confirmed that the molecular mass of the AKTIP was approximately 22 kDa (Figure S2), which was consistent with the predicted molecular mass. It also showed that the protein expression of AKTIP was downregulated after RNAi at the highest dsRNA concentration (Figure 3B).

### 2.4. Effects of NlAKTIP RNAi on Body Weight and Size

The body weight and size of BPH were examined at day 7 after the dsAKTIP treatment was initiated. The results showed that BPH fed on dsAKTIP had disrupted growth, showing lower body weights and smaller sizes compared to the untreated and GFP controls. With increases of dsAKTIP concentrations, the average body weight of treated BPH decreased to 1.36 mg (0.02 μg/μL dsAKTIP), 1.34 mg (0.1 μg/μL dsAKTIP treatment) and 0.77 mg (0.5 μg/μL dsAKTIP treatment), which were significantly lower than the body weights of the untreated (2.21 mg) and the GFP (1.74 mg) control groups (Figure 3C). There was a significant difference between the dsAKTIP treated groups and the untreated control, while there was no significant differences between the untreated and GFP controls. Moreover, with concentrations of dsAKTIP increasing, the body weight and size tended to decrease, indicating that the growth of BPH was seriously inhibited due to knockdown of the target gene (Figure 3D).

### 2.5. Effects of NlAKTIP RNAi on Eclosion and Ovarian Development

**Figure 4:**
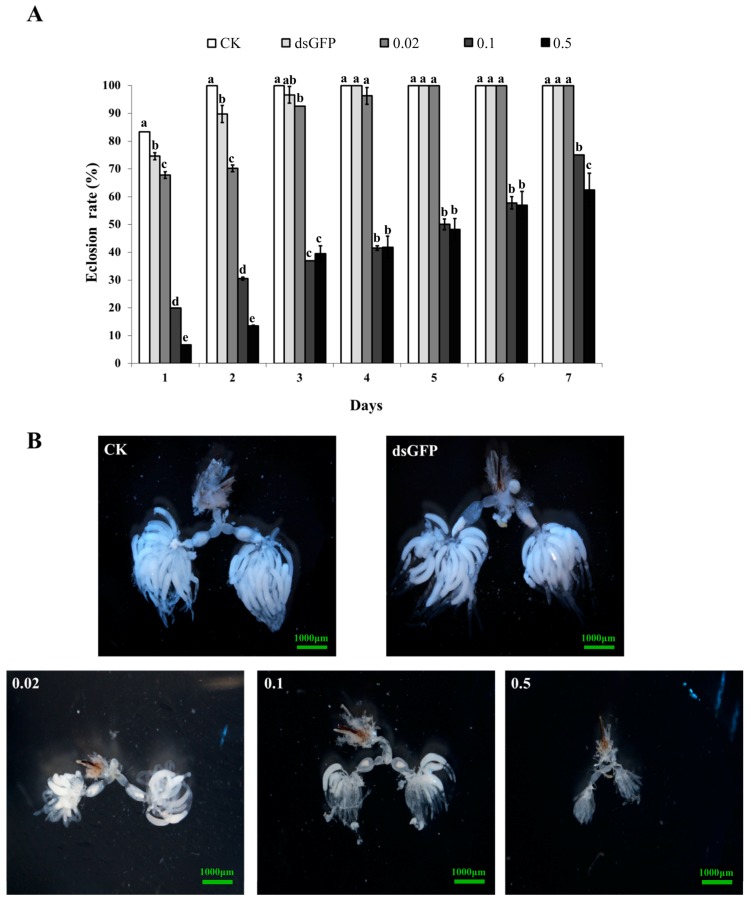
The effect of *NlAKTIP* RNAi on BHP eclosion and ovary development. CK: female fed on D-97 (untreated diet); dsGFP: female fed on 0.5 μg/μL dsGFP control; 0.02: female fed on 0.02 μg/μL dsAKTIP; 0.1: female fed on 0.1μg/μL dsAKTIP; 0.5: female fed on 0.5 μg/μL dsAKTIP. (**A**) The eclosion rate of BPH. The results were from the same bugs measured over time, and expressed as the means ± SD from 20 individuals with three replications. The eclosion rate was compared by Tukey’s test at each sampling time. Different letters of a, b, c, d and e on the bars indicate significant differences among treatments (*p* < 0.05); (**B**) The effect of *NlAKTIP* RNAi on ovarian development.

At day 1, the BPH in the untreated control, GFP control, and 0.02 μg/μL dsAKTIP low concentration treatment groups showed relatively high eclosion rates of 83.3%, 74.6% and 67.8%, respectively. All BPH individuals in these three treatments completed eclosion within 5 days. However, the eclosion rate in the medium (0.1 μg/μL dsAKTIP) and high (0.5 μg/μL dsAKTIP) concentration treatment groups was very low (not more than 20%) at day 1. This lower eclosion rate lasted for at least 7 days and was significantly lower than the eclosion rates of the untreated and GFP controls, as well as those of the low concentration treatment groups (*p* < 0.05). These results indicated that the medium concentration dsRNA treatment was enough to achieve low eclosion rate (Figure 4A).

Ovarian anatomy was evaluated at day 7, and the results showed that the BPH ovaries in the groups of untreated control, GFP control and low concentration treatment were all well developed with a state of grade IV, and the BPH ovaries in the medium treatment group were at grade III, while the BPH ovaries in the high concentration treatment group were seriously inhibited at grade I (Figure 4B). These results showed that the inhibition of ovary development by RNAi was concentration-dependent.

### 2.6. Effects of NlAKTIP RNAi on BPH Survival Rates

**Figure 5:**
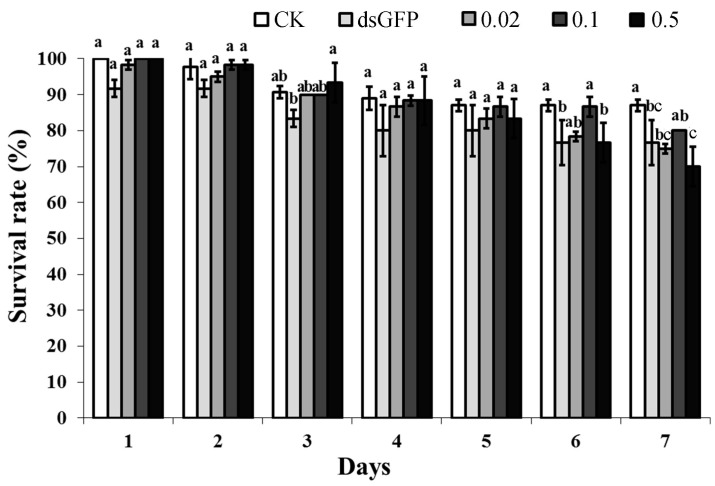
Survival rate of the different groups fed with RNAi. CK: female fed on D-97 (untreated diet); dsGFP: female fed on 0.5 μg/μL dsGFP control; 0.02: female fed on 0.02 μg/μL dsAKTIP; 0.1: female fed on 0.1 μg/μL dsAKTIP; 0.5: female fed on 0.5 μg/μL dsAKTIP. The results were from the same bugs measured over time, and expressed as the means ± SD from 20 individuals with three replications. The eclosion rate was compared by Tukey’s test at each sampling time. Different letters of a, b and c on the bars indicate significant differences among treatments (*p* < 0.05).

Generally, the survival rates of BPH decreased after continuous feeding with an artificial diet, but there is no obvious difference between BPH groups treated with dsAKTIP and CK/dsGFP, except at day 6 and day 7. Moreover, the survival rate of the BPH groups still remained at a high level (mostly over 80%), indicating that the knockdown of the target gene *NlAKTIP* did not result in high mortality (Figure 5).

## 3. Discussion

In the past several decades, great efforts have been made to explore the mechanisms of BPH adaptation to resistant rice varieties, including ecological, physiological and molecular aspects, but it still remains largely unknown how the resistant rice suppresses the growth and development of BPH [2,24]. In our current study, we found that *NlAKTIP* was down-regulated when the BPH was reared on resistant rice variety RHT, including different colonies of RF (RF1 to RF5) and Rh colony, suggesting that *NlAKTIP* might be an important responsive gene in BPH fed on resistant rice. To reveal possible involvement of *NlAKTIP* in interactions between BPH and resistant rice, we suppressed the expression of *NlAKTIP* by RNAi and found significantly decreased growth and development of BPH. We found that the treated BPH showed lower body weights, delayed eclosion and slower ovarian development. Such phenotypical changes are similar to those found when BPH are fed on resistant rice. Peñalver Cruz *et al*. [25] also found that development of BPH from the susceptible variety TN1 showed decreased biomass and delayed growth during adaptation to the resistant variety RHT. It is expected that dsRNA treated smaller insects will ingest less phloem sap and do less harm to rice. In addition, delayed eclosion and suppressed ovary development will result in a smaller population of BPH. An exciting implication of our finding is the pervasive connection of AKTIP/AKT to BPH growth and development. Nevertheless, further research to clarify the exact role of *NlAKTIP* in interactions between BPH and resistant rice varieties would be necessary before it can be used as a new target with which to develop novel strategies for controlling BPH.

The insulin/PI3K/AKT pathway was found to have a role in controlling the size and growth of tissues [26]. In a previous gene expression profiling study [23], genes encoding for insulin and the insulin receptor were also decreased in the BPH colonies reared on RHT when compared with the Tn colony, indicating that the suppression of the insulin/AKT signaling pathway should relate to the delay of BPH growth (Figure S3). AKT has been demonstrated to play a central role in a number of cellular responses to growth factors and insulin [22]. AKT mediates most of the growth-promoting actions of the insulin/PI3K/AKT signaling pathway through the phosphorylation of several substrates that affect cell growth, cell cycle entry, and cell survival [14,26]. Since activated AKT induces protein synthesis via mTOR and downstream elements, the down regulation of *NlAKTIP* or inactivation of AKT should be able to inhibit various processes and delay the development of BPH. In our study, downstream molecules such as mTOR and S6K were also down-regulated during the interaction of BPH and the resistant varieties. At the same time, a ribosomal protein S6 in the Rh colony upregulated, likely to compensate for the inhibition from the upstream (Figure S3). This result suggests that the AKT/mTOR signaling pathway takes part in regulating the growth and development of BPH, probably by affecting the protein synthesis as described [27].

Suppression of the insulin signaling pathway appears to induce physiological responses that enhance resistance to diverse forms of stress [28,29,30]. For example, in *Caenorhabditis elegans*, starvation can induce adult reproductive diapause, which enables sexually mature adults to delay reproductive onset and extend total adult life span [31]. Similarly, ILP-1(ins-1) induces dauer arrest under low food levels [32]. When exposed to the resistant variety RHT, a BPH generally ingests a lower quantity of phloem sap, which means that the BPH is, in fact, experiencing starvation [25]. Therefore, the question arises of whether this stress (starvation or suppressing of *NlAKTIP*) can increase the resistance of BPH to the resistant rice. To test this, we conducted an experiment by knocking-down the *NlAKTIP* gene of susceptible Tn insects and then placing them on a diet of the resistant rice variety RHT, but the results showed that suppression of this gene did not enhance the adaptability of BPH. Therefore, it is most likely that while *NlAKTIP* was, in fact, suppressed by resistant rice, this does contribute to host adaptation. From this point of view, the *NlAKTIP* gene has some potential to be a target to consider for BPH control. It might be better to combine this *NlAKTIP* gene with some other gene which effectively suppresses the growth and survival of BPH, such as NlPIK3R1 cloned by our group [33], so that we can achieve a higher RNAi efficiency by suppressing both the growth and the survival of BPH in the field. Some practicable ways of using RNAi in the field to control BPH may be spraying or irrigating by dissolving dsRNA into the water. In fact, the ability to produce kilogram quantities of dsRNA has been developed, which has made it possible to produce enough dsRNA for field applications at low cost [34].

## 4. Experimental Section

### 4.1. Insects

Insects were collected from the following BPH colonies: Tn colony (continuously reared on susceptible rice variety TN1 for more than 50 generations), Rh colony (continuously reared on resistant rice variety RHT for more than 50 generations), and five RF colonies (Tn colony exposure to resistant RHT for 1, 2, 3, 4, and 5 generations, respectively). The rearing conditions were 26 ± 2 °C, relative humidity 80% ± 5% and a 16 h light/8 h dark photoperiod.

### 4.2. Cloning of Full-Length NlAKTIP cDNA

Total RNA was extracted from the BPH samples at different stages using Trizol. RNA concentrations were determined using Nanodrop 2000 (Thermo, Wilmington, DE, USA). Primers (Table 1) for RACE were designed according to our previous cDNA sequence information from the BPH transcriptome. The 5′ and 3′ RACE was conducted using the BD SMART™ RACE cDNA Amplification Kit (Clontech, Palo Alto, CA, USA). The PCR products were examined by electrophoresis in a 1% agarose gel and stained with ethidium bromide. The selected PCR products were purified with the AxyPrep™ DNA Gel Extraction Kit (Axygen Scientific Inc., Union, CA, USA), cloned into the pMD18-T vector (TaKaRa, Tokyo, Japan) and sequenced by the Sunny Company (Shanghai, China). Sequences from 3′ and 5′ RACE as well as the core sequence were spliced together to obtain the full length cDNA of *NlAKTIP*, which was then verified by PCR with AKTIP-FL-F and AKTIP-FL-R primers (Table 1) designed at both ends of the spliced sequence.

**Table 1:** Primers used in cDNA sequencing, RT-qPCR analysis and dsRNA synthesis of the *NlAKTIP.*

Primer Name	Sequence (5′→3′)
cDNA cloning	
3′RACE outer	TCAAACGGCAAGGCTCAT
3′RACE inner	CGTCCACAACAATCTTCTTCGC
5′RACE outer	AGGAGAAGAAGCGGAGGG
5′RACE inner	GGCATAACATAGAGCCCTGGAA
AKTIP-FL-F	ACATGGGAGACAATAACACATAACC
AKTIP-FL-R	CGACTTTAAAGCTTCTCTTGCTGTC
RT-qPCR	
QAKTIP-F	TGTCTGCCAAAATGATCGAAC
QAKTIP-R	CTCCATGATAGAGCCCTT
Actin-F	TGCGTGACATCAAGGAGAAGC
Actin-R	CCATACCCAAGAAGGAAGGCT
dsRNA synthesis	
dsAKTIP-F	GGATCC$\underset{\sim }{\text{T}}$$\underset{\sim }{\text{A}}$$\underset{\sim }{\text{A}}$$\underset{\sim }{\text{T}}$$\underset{\sim }{\text{A}}$$\underset{\sim }{\text{C}}$$\underset{\sim }{\text{G}}$$\underset{\sim }{\text{A}}$$\underset{\sim }{\text{C}}$$\underset{\sim }{\text{T}}$$\underset{\sim }{\text{C}}$$\underset{\sim }{\text{A}}$$\underset{\sim }{\text{C}}$$\underset{\sim }{\text{T}}$$\underset{\sim }{\text{A}}$$\underset{\sim }{\text{T}}$$\underset{\sim }{\text{A}}$GGGAGAGCTCTATGTTATGCCCTCCG
dsAKTIP-R	GGATCC$\underset{\sim }{\text{T}}$$\underset{\sim }{\text{A}}$$\underset{\sim }{\text{A}}$$\underset{\sim }{\text{T}}$$\underset{\sim }{\text{A}}$$\underset{\sim }{\text{C}}$$\underset{\sim }{\text{G}}$$\underset{\sim }{\text{A}}$$\underset{\sim }{\text{C}}$$\underset{\sim }{\text{T}}$$\underset{\sim }{\text{C}}$$\underset{\sim }{\text{A}}$$\underset{\sim }{\text{C}}$$\underset{\sim }{\text{T}}$$\underset{\sim }{\text{A}}$$\underset{\sim }{\text{T}}$$\underset{\sim }{\text{A}}$GGGAGAGATGCTTCTTGGTTGACTGC
dsGFP-F	GGATCC$\underset{\sim }{\text{T}}$$\underset{\sim }{\text{A}}$$\underset{\sim }{\text{A}}$$\underset{\sim }{\text{T}}$$\underset{\sim }{\text{A}}$$\underset{\sim }{\text{C}}$$\underset{\sim }{\text{G}}$$\underset{\sim }{\text{A}}$$\underset{\sim }{\text{C}}$$\underset{\sim }{\text{T}}$$\underset{\sim }{\text{C}}$$\underset{\sim }{\text{A}}$$\underset{\sim }{\text{C}}$$\underset{\sim }{\text{T}}$$\underset{\sim }{\text{A}}$$\underset{\sim }{\text{T}}$$\underset{\sim }{\text{A}}$GGGATACGTGCAGGAGAGGAC
dsGFP-R	GGATCC$\underset{\sim }{\text{T}}$$\underset{\sim }{\text{A}}$$\underset{\sim }{\text{A}}$$\underset{\sim }{\text{T}}$$\underset{\sim }{\text{A}}$$\underset{\sim }{\text{C}}$$\underset{\sim }{\text{G}}$$\underset{\sim }{\text{A}}$$\underset{\sim }{\text{C}}$$\underset{\sim }{\text{T}}$$\underset{\sim }{\text{C}}$$\underset{\sim }{\text{A}}$$\underset{\sim }{\text{C}}$$\underset{\sim }{\text{T}}$$\underset{\sim }{\text{A}}$$\underset{\sim }{\text{T}}$$\underset{\sim }{\text{A}}$GGGCAGATTGTGTGGACAGG

Underlined sequences represent for protective bases, and wavelined sequences represent for T7 promoter.

The sequences of the 17 different organisms in Figure 1 were used to construct a phylogenetic analysis. ClustalX was used to align the protein sequences with the homologous genes. A phylogenetic tree was constructed by MEGA 6.0 using the neighbor-joining analysis with 1000 bootstrap replicates.

### 4.3. Gene Expression Analysis of NlAKTIP

Two different sets of BPH colonies were prepared for gene expression pattern analysis. The first set included BPH samples from the Tn and Rh colonies at various developmental stages. Based on the results of first set that showed that the gene was highly expressed in gravid females, the second set only consisted of gravid females of Tn, RF1, RF2, RF3, RF4, RF5, and Rh colonies. The expression of *NlAKTIP* mRNA was quantified using RT-qPCR. The experiment was performed on 3 independent occasions. Ten adults or 30 to 50 nymphs were combined for RNA extraction, and 1 μg total RNA was used as templates for synthesizing cDNA using the PrimeScript RT Reagent Kit with gDNA Eraser (Takara, Japan). Gene-specific primers for QAKTIP-F and QAKTIP-R (Table 1) were designed for RT-qPCR according to the complete cDNA sequence of *NlAKTIP*, and the β-actin gene was used as an internal control [35]. The reaction volume was 10 µL, and the cycling conditions were as follows: (i) 95 °C for 3 min; (ii) 95 °C for 45 s; (iii) 58 °C for 30 s; and (iv) repeat steps (ii) and (iii) for 40 cycles. Real-time data were collected using the IQ™5 Multicolor RT-qPCR detection system (Bio-Rad, Hercules, CA, USA). The relative expression level was calculated using 2^−ΔΔ*C*t^ method [36]. The Tn colony was used as the reference (1–2 instar nymphs for first set, and gravid female for second set). All the mRNA expression data were presented as the means ± SE of three technical replicates and a Student’s *t*-test was used to analyze the data.

### 4.4. Synthesis of dsRNA

The cDNA for the dsAKTIP synthesis was amplified using the primers provided in Table 1, and cloned into the pMD18-T vector (TaKaRa, Tokyo, Japan), transferred into JM109 and sequenced. To improve the efficiency of the transcription, the primers were designed according to a previous method [37] in which the protecting bases GGATCC and T7 promoter (TAATACGACTCACTATA) were added to the 5′ ends of the specific primers, respectively. The clone with the T7 promoter sequences was selected, propagated, and plasmids extracted. Using MEGAscript^®^ T7 High Yield Transcription Kit (Ambion, Austin, TX, USA) to synthesize dsRNA according to the manufacturer’s instructions, the dsAKTIP was amplified between the 453 and 769 bp sites of the full-length cDNA of *NlAKTIP*. Using the same method, dsGFP located between the 281 and 630 bp sites of the GFP cDNA was prepared, according to the sequence of a green fluorescent protein GFP (GenBank access no: AF234298). The dsAKTIP and dsGFP fragments did not contain the fragments for RT-qPCR and thus would not affect the RNAi results. The amplification reactions consisted of 40 cycles of 95 °C for 30 s, 70 °C for 30 s, 72 °C for 45 s, and a final extension step of 72 °C for 10 min. The PCR products were verified by sequencing. Only the sequences that were perfectly aligned (100%) were used as templates for dsRNA synthesis and the following RNA interference experiments.

### 4.5. Insect Bioassays 

For RNA interference, the dsRNA was added to the artificial D-97 diet [38], which was fed to BPH according to a previous method [38], with some modifications. Briefly, the 20 μL artificial D-97 diet, either with or without dsRNA, was placed in a feeding device and enclosed between two layers of stretched Parafilm M that covered both ends of the glass chamber. The artificial diet was renewed every day. The concentrations of dsRNA tested were 0.02 μg/μL (low concentration treatment), 0.1 μg/μL (medium concentration treatment) and 0.5 μg/μL (high concentration treatment). The artificial D-97 diet only was used as the untreated control, and D-97 + 0.5 μg/μL dsGFP was used as the dsRNA treated control.

About 7000 individuals of two-day-old (2nd instar) BPH nymphs on the TN1 variety were first moved to the D-97 artificial diet system without dsRNA for pre-feeding. To ensure the RNAi treatment could cover the whole stage of gravid females, during which the target gene was highly expressed, dsRNA was added into the D-97 artificial diet when these nymphs grew to 5th instar. Then, 20 females were collected for RNAi treatments with three replications per treatment. When the untreated control began to eclose, the survival and eclosion rate were recorded daily for 7 days. During this period, the same insects were measured over time. On day 7, the BPH in each treatment were firstly weighed (Sartorius BSA224S, Hamburg, Germany) and then dissected to examine the development of the ovary according to the method of Dong *et al.* [39]. Photographs were taken under a stereoscope equipped with a digital image acquisition system (Nikon SMZ1500, Tokyo, Japan).

A parallel experiment was performed to analyze the mRNA expression level. When the untreated control began to eclose, three female adults were selected randomly for subsequent RNA extraction at days 1, 3, 5 and 7 after eclosion. The RT-qPCR methods, primers and reaction conditions were as described above. The insects were maintained at 26 ± 2 °C, relative humidity 80% and a 16 h light/8 h dark photoperiod.

### 4.6. Antibody

A peptide (PFKRQGSLRKVLPPC) from the NlAKTIP protein was synthesized, and rabbits were immunized with this peptide to obtain the polyclonal antibody to NlAKTIP by GenScript Inc. (Nanjing, China).

### 4.7. Western Blot Analysis

At day 7, three female BPH were homogenized in 1× PBS followed by adding 2× SDS sample buffer was added. The lysates were boiled for 5 min, followed by centrifugation at 10,000× *g* for 5 min. Ten microliters of each sample was loaded onto the SDS gels. After electrophoresis, the proteins were transferred to polyvinylidene difluoride membranes. The blots were then blocked in TBST (0.1% Tween 20 in TBS) and 5% nonfat powdered dry milk (*w*/*v*) for 2 h at room temperature. The blot was then incubated for 12 h at 4 °C in a TBST solution containing the primary antibody against NlAKTIP at a dilution of 1:1000 to probe NlAKTIP. The membrane was washed five times with TBST for 5 min each time and then incubated in a 1:1000 dilution of the horseradish peroxidase-linked secondary antibody (Solarbio, Beijing, China). The membrane was washed five times, the immunoreactivity was detected using the DAB Horseradish Peroxidase Color Development Kit (Sangon Biotech, Shanghai, China) and the membrane was imaged with a GS-800 Calibrated Densitometer (Bio-Rad, Hercules, CA, USA).

### 4.8. Data Analysis

The data of gene expression, eclosion rate and survival rate from RNAi experiments was analyzed by Tukey’s test at each sampling time. However, data of gene expression from different BPH colonies and average body weights from RNAi experiments was analyzed by method of Student’s *t*-test.

## Supplementary Materials

Supplementary materials can be found at http://www.mdpi.com/1422-0067/16/09/22888/s1.
