# Supplementary material for: Roles of NlAKTIP in the Growth and Eclosion of the Rice Brown Planthopper, Nilaparvata lugens Stål, as Revealed by RNA Interference
Source: Int J Mol Sci. 2015 Sep 22;16(9):22888–903. doi: 10.3390/ijms160922888 (PMC4613341; doi:10.3390/ijms160922888)
Supplement: Supplementary file 1 [file ijms-16-22888-s001.pdf]

Supplementary Information

```
1      acatgggagacaataacacataacctcaaaaaatcaatatgaagctcaaaataagtattaactaatttgtttactact
79     atctttaaaaacttcgttacaaataataaaaatattcatagcttggagttatctagaaaaatcttagcaaataagaga
157    gtttgtagaatttgaggtaatcaatcaagtaaactgtgtcATGGCTTCAGGTAGTTTCAAAAAGGGTTCTGAGGCAAC
1                                              M  A  S  G  S  F  K  K  G  S  E  A  T
235    AGAGACAAATGAACCTTTCAAACGGCAAGGCTCATTGCGAAAAGTTCTTCCTCCTAAACAATATGGAGAATCAATGCT
14      E  T  N  E  P  F  K  R  Q  G  S  L  R  K  V  L  P  P  K  Q  Y  G  E  S  M  L
313    TAGCATGTCTGCCAAAATGATCGAACGTCCACAACAATCTTCTTCGCAAACAAGTCTTTACAAGCAAGCTTACAGTCC
40      S  M  S  A  K  M  I  E  R  P  Q  Q  S  S  S  Q  T  S  L  Y  K  Q  A  Y  S  P
391    ATTCTTCCAAGAGTATAGTATAATGAGTGAATACAACCTACTGACTAAGAAATGTCTTCCAGGGCTCTATGTTATGCC
66      F  F  Q  E  Y  S  I  M  S  E  Y  N  L  L  T  K  K  C  L  P  G  L  Y  V  M  P
469    CTCCGCTTCTTCTCCTTTATTATGGTTTGGTGTATTGTTCATCAAAAAAGGGCTCTATCATGGAGGTATCTTTCGTTT
92      S  A  S  S  P  L  L  W  F  G  V  L  F  I  K  K  G  L  Y  H  G  G  I  F  R  F
547    CAATTTGGAAACTCCCGAGACCTTTCCTGATTGCACATGTCCGAAAGTTGTATTCGAGTCTAAAGTCTTCCATCCTAG
118     N  L  E  T  P  E  T  F  P  D  C  T  C  P  K  V  V  F  E  S  K  V  F  H  P  S
625    TATAGACATTGCAACTGGCGAAGTTTTCTTGAATCAAACATTTCCGGAGTGGAAGAAAGACGTGAACCATTTATGGCA
144     I  D  I  A  T  G  E  V  F  L  N  Q  T  F  P  E  W  K  K  D  V  N  H  L  W  Q
703    AATACTTGAACACGTATTGCAGTTGTTTCTCAGCATTGACGTCAAGGATGCAGTCAACCAAGAAGCATCTATCTCTAT
170     I  L  E  H  V  L  Q  L  F  L  S  I  D  V  K  D  A  V  N  Q  E  A  S  I  S  M
781    GTGAacgatatggatgccttcgtcaaccgagtgaaagagagtgtagactcttctcagaataatttgttcgacgaaccg
196     *
859    acgtcaagtgatcctactatctccgattcaatcaatatgatgaaaccctccataagacagcaagagaagctttaaagt
937    cgaaaaaaaaaaaaaaaaaaaaaaaaaa
```

Figure S1. The nucleotide and deduced amino acid sequences of *NlAKTIP*. The deduced amino acid sequence is shown below the cDNA sequence. The start and stop codons are shown in boldface, and an asterisk represents the position of the stop codons.

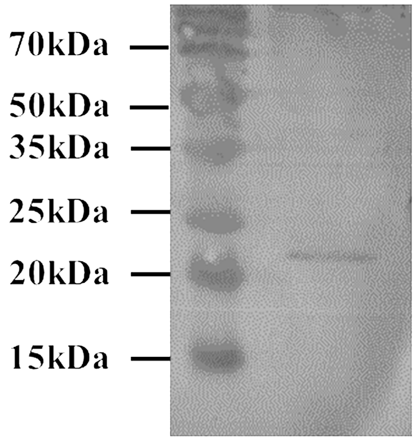

Figure S2. Test of the AKTIP antibody specificity. The polyclonal rabbit antibody is generated by PolyExpress™ Antibody Service.

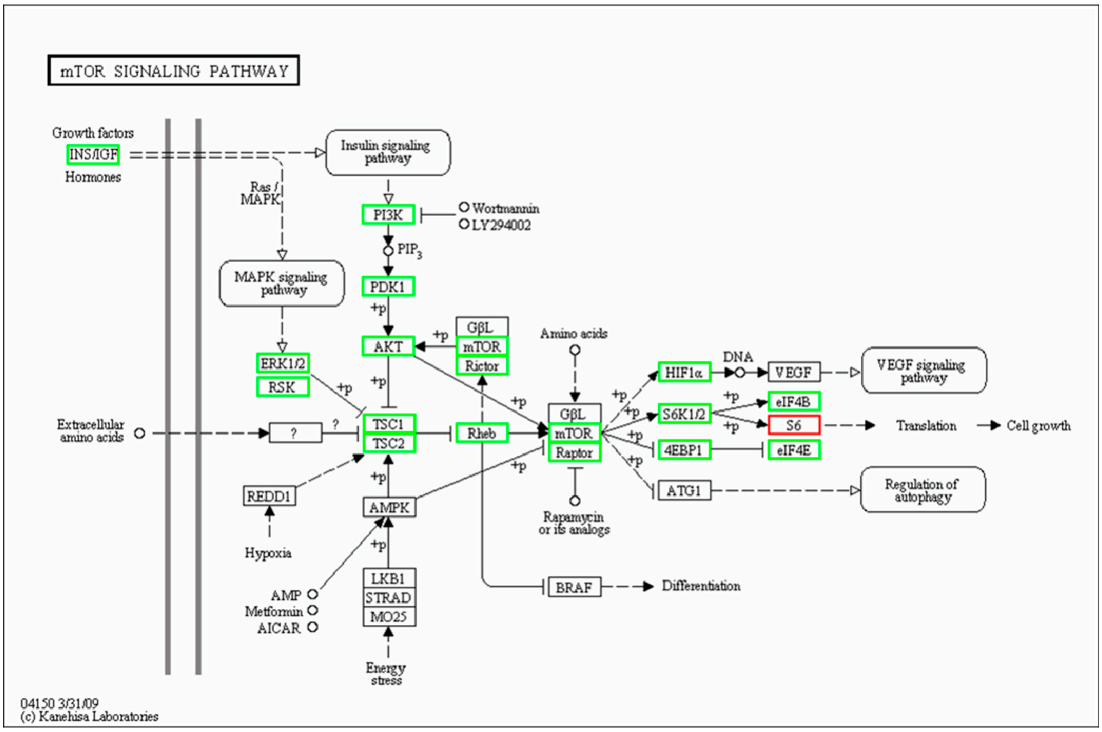

Figure S3. Gene expression profile of the insulin/AKT/mTOR signaling pathway in BPH. Boxes highlighted in green correspond to down-regulated genes, while the red box indicates up-regulated genes in the Rh colony compared with the Tn colony. Solid line and black arrows represent for directly stimulatory modification; Dotted line with black arrow represent for indirectly stimulatory modification; Dotted line with white arrow represent for translocation.
